# Promising high fidelity genetic markers for sexing *Cannabis sativa* seedlings

**DOI:** 10.1093/g3journal/jkaf077

**Published:** 2025-04-09

**Authors:** Djivan Prentout, Salma El Aoudati, Fabienne Mathis, Gabriel A B Marais, Hélène Henri

**Affiliations:** Laboratoire de Biométrie et Biologie Évolutive UMR 5558, Université de Lyon, Université Lyon 1, CNRS, Villeurbanne F-69622, France; Department of Biological Sciences, Columbia University, New York, NY 10027, USA; Laboratoire de Biométrie et Biologie Évolutive UMR 5558, Université de Lyon, Université Lyon 1, CNRS, Villeurbanne F-69622, France; Hemp-it ADN, R&D Department, Beaufort en Anjou 49 250, France; Laboratoire de Biométrie et Biologie Évolutive UMR 5558, Université de Lyon, Université Lyon 1, CNRS, Villeurbanne F-69622, France; Laboratoire de Biométrie et Biologie Évolutive UMR 5558, Université de Lyon, Université Lyon 1, CNRS, Villeurbanne F-69622, France

**Keywords:** *Cannabis sativa*, sex identification, PCR, Y chromosome, Plant Genetics and Genomics

## Abstract

The uses of *Cannabis sativa*, a dioecious species with an XY sex chromosome system, are varying from fiber and oil to cannabinoids, among others. In most cases, males are undesirable, and the sexual dimorphism at immature plants is too subtle for reliable phenotypic sexing, making genetic approaches promising. In this technical note, we present a multiplex PCR mix that includes 2 markers of Y-specific coding regions and 1 autosomal control marker. This PCR mix, tested across 12 hemp-type cultivars, encompassing approximately 200 individuals, achieved a 99.5% true positive rate in identifying the sex of *C. sativa* samples. Despite being tested on a limited number of germplasm, we believe that these markers offer a promising solution for sexing a larger diversity of *C. sativa* seedlings.

## Introduction

*Cannabis sativa* is one of the oldest domesticated plants and is used in a wide range of purposes. Two primary groups of cultivars have been described: hemp types and drug types (Rehman *et al*. 2021; Ren *et al*. 2021). Hemp-type cultivars are typically used for textiles, food or oilseed production, whereas drug types are cultivated for medicinal and recreational uses (Rehman *et al*. 2021). The recent global legalization of the latter applications has led to a significant increase of the drug-type production in the past decade. As a consequence, the global economy of *C. sativa* now surpasses tens of billions of dollars annually (Malabadi *et al*. 2024).

In nature, *C. sativa* is dioecious (i.e. male and female flowers are on separate individuals), and the sex is determined by an XY pair of sex chromosomes (Ming *et al*. 2011; Divashuk *et al*. 2014). Female plants are preferred for most of the purposes listed before. For example, female plants produce thicker fibers and are therefore favored for textile production. More critically, males do not produce cannabinoids (THC and CBD) in high concentration, and in case of a pollination, females cease their production (Chandra *et al*. 2017). This motivates an early identification of males in crops, and more generally, a better control of the sex in *C. sativa* (Chandra *et al*. 2017).

Three primary strategies can be employed to remove male plants from a crop (1) sex reversal of the individuals, (2) vegetative propagation (i.e. cloning) of females, or (3) identification, and withdrawing, of male individuals from the crops. Sex reversal typically implies the administration of a silver agent to females, in order to produce hermaphrodite flowers (see Chandra *et al*. 2017). Such hermaphrodites have 2 X chromosomes, leading their self-pollination to produce only “feminized” seeds (with a true positive rate of 95%; see Chandra *et al*. 2017). However, besides being pricey, this process is unstable throughout generations (reviewed in Chandra *et al*. 2017; Malabadi *et al*. 2023). Vegetative propagation of female individuals requires highly controlled conditions to avoid sex plasticity. Additionally, somatic mutations may accumulate over generations, decreasing disease resistance or cannabinoid content (Trancoso *et al*. 2022). Phenotypic identification of the sex in immature *C. sativa* plants is nearly impossible due to the lack of sexual dimorphism in young individuals. Genetic approaches offer a promising alternative. Recently, a sex marker utilizing a PACE-PCR Allele Competitive Extension showed a high success in sex identification (Toth *et al*. 2020). However, this marker, along with previous markers, is located in retrotransposons, which may affect its accuracy across different cultivars (Törjék *et al*. 2002; Sakamoto *et al*. 2005; Toth *et al*. 2020).

In a previous analysis, we conducted a transcriptome-wide segregation analysis of *C. sativa* to identify genes located in the nonrecombining region of the sex chromosomes (hereafter referred to as sex-linked genes; Prentout *et al*. 2020). We identified more than 550 sex-linked genes and showed that some regions of the sex chromosomes are highly divergent (X-Y synonymous divergence reaches 40%) (Prentout *et al*. 2020). These highly divergent regions are of particular interest because they could be shared in all *C. sativa* populations/cultivars, providing a unique opportunity to develop universal Y-linked markers for an early sex identification. In the present study, we use these divergent sex-linked genes to develop a multiplex PCR assay aimed at identifying males in *C. sativa* seedlings.

## Material and methods

### Design and in silico validation of the primers

Of the 565 sex-linked genes identified in Prentout *et al*. (2020), we selected the 347 genes where the Y copy remains expressed. From this subset, we kept the genes that meet the following 2 criteria: (1) they are located in the oldest stratum of the sex chromosomes (Prentout *et al*. 2020, 2021), and (2) they are among the 20 pairs of XY genes with the highest synonymous divergence (*dS*). Finally, we filtered out genes with an intronic size of <300 bp. For each gene pair, we aligned the Y-linked sequence from the cross we generated (cultivar Zenitsa, hemp-type; see Prentout *et al*. 2020) with 3 X-linked sequences: (1) one from the same cross as the Y sequence, (2) one from a Purple Kush cultivar (drug-type), and (3) one from a Finola cultivar (hemp-type) (the 2 latter from van Bakel *et al*. 2011). This increased the probability of identifying X-Y divergent sites shared by different populations. We aligned the sequences with the Qiagen CLC Main workbench tool “create alignment” (version 8.0.1; see https://www.qiagenbioinformatics.com) and used the tool “Design Primer” (Krämer *et al*. 2014). We kept the coding regions with at least 2 fixed differences between X-linked and Y-linked sequences and avoided complementary forward and reverse primers to avoid association between our primers during PCR.

We used the in silico PCR tool from the van Bakel Lab to test the size of the amplicons and to verify that a unique region of the genome was amplified (http://genome.ccbr.utoronto.ca/). Our primers were tested in silico on both the Purple Kush and the Finola genomes. As both genomes are female genomes, we expected an amplification of X-linked primers but not of the Y-linked.

### Plant material for in vitro validation

We collected 192 samples from 12 hemp-type cultivars. The sampling included both accessions and varieties. Seeds were sown in a climatic chamber and exposed to 18 h of light and 6 h of darkness, at 22°C and 18°C, respectively. Flowering was induced by altering the light cycle to 12 h of daylight, with the same temperature conditions as the initial cycle. Once the plants could be sexed phenotypically, we collected 8 leaf discs per sample and air-dried them for DNA isolation.

### DNA extraction

We used the CTAB method from Koh *et al*. (2021) protocol. From 10 to 30 mg of leaves were placed in a 2-mL round bottom tube, with a 5-mm stainless steel bead, and placed for at least 15 min at −80°C. Frozen samples were ground into powder by setting the Tissue Lyser (Retsch) speed at 20 Hz for 2 min. Four hundred microliters of CTAB DNA extraction buffer (2% *w*/*v* CTAB, 20 mM EDTA.Na₂.2H₂O, 1.4 M sodium chloride, and 100 mM Tris) was then added to the tube supplemented with 0.3% (*v*/*v*) β-mercaptoethanol. Tubes were incubated at 65°C for 30 min in a thermomixer (Eppendorf) under agitation at 400 rpm. Debris was separated from the liquid part through centrifugation (15,000 × *g* for 5 min at room temperature). The resulting supernatant (270 µL) was transferred to a new 1.5-mL tube, and an equal volume of 24:1 chloroform:isoamyl alcohol was added. The tubes were constantly inverted for 5 min to mix the solution, and the aqueous phase was separated by centrifugation (15,000 × *g* for 5 min at 4°C). Two hundred forty microliters of the recovered aqueous phase was transferred to a new 1.5-mL tube, and RNA was digested with the addition of 0.5 μL of 10 mg/mL of RNAseA and incubated for 15 min at 37°C. Chloroform:isoamyl alcohol extraction was repeated, and 240 μL of the resulting supernatant was transferred to a new tube. DNA was precipitated by the addition of ½ volume (120 μL) of 5 M NaCl and 3 volumes (720 μL) of ice-cold absolute ethanol and incubation at −20°C for at least 30 min. Tubes were centrifuged at 15,000 × *g* for 5 min at 4°C to pellet the DNA precipitates. Pellets were washed twice with 500 μL of 70% ethanol (centrifuged at 15,000 × *g* for 5 min at 4°C) and resuspended in 50 μL of nuclease-free water.

### PCR reaction

The DNA extracts were diluted 1:10 before being used for the PCR reactions. PCR reactions were performed in a final volume of 10 µL using 5 µL of Taq Ozyme HS mix 2× (Ozyme, ref #OZYA006), 1.5 µL of primer mix comprising 10 µM of each primer, 1.5 µL of ultrapure water, and 2 µL of 1:10 diluted DNA. The cycling conditions consisted of an initial denaturation step lasting 1 min at 95°C, followed by 35 cycles of denaturation at 95°C for 15 s, annealing at 58°C for 15 s, and elongation at 72°C for 30 s, with a final extension step at 72°C for 5 min. The PCR results were revealed through an agarose gel electrophoresis.

## Results

We selected 2 Y-linked primers and 1 autosomal primer in order to develop a multiplex approach (see *Materials and methods*; Table 1). The autosomal primer is used to control if the PCR worked, and the 2 Y-linked primers increase the true positive rate for distant cultivars. This protocol was tested on 192 samples (96 males and 96 females) from 12 different hemp cultivars (Table 2). It is worth noting that the number of samples tested for each cultivar is ranging between 1 and 33 (Table 2). Among the 192 tested samples, the sex was correctly inferred for 191 of them, meaning a true positive rate of 99.5%. Only 1 female individual was detected as a male (bold number in Table 2).

**Table 1.** Primer IDs and sequences along with the gene ID they belong to, the segregation type, the XY divergence, and the amplicon length.

	Forward sequence	Reverse sequence	Gene ID	XY dS	Amplicon length
Primer 23 (XY)	GTTATATGGACATGGACTCT	TTTTTCTGATAGTGCGCC	PK05076.1	0.225	174 bp
Primer 36 (XY)	AAAAGGGGTGAAAAGGTG	CCTCTCTTTCTTGATGAACT	PK18889.1	0.187	300 bp
Primer 9 (autosomal)	GTTTCATCGGTTGCTTTCT	TCCCTATTCTCATCCCTCT	PK01688.1	NA	514 bp

**Table 2.** Number of samples tested for each of the 12 cultivars.

Cultivar	Number of sample	Male sexed as male	Female sexed as female	Male sexed as female	Female sexed as male	Experiment failed	True positive success rate (%)
Pu	22	10	12	0	0	0	100
Cr	30	15	14	0	**1**	0	97
Ch	8	4	4	0	0	0	100
D1	33	15	18	0	0	0	100
Ti	18	12	6	0	0	0	100
Ma	12	6	6	0	0	0	100
Ks	29	15	14	0	0	0	100
D8	29	15	14	0	0	0	100
D2	1	0	1	0	0	0	100
Ca	1	0	1	0	0	0	100
No	1	0	1	0	0	0	100
Fi	8	4	4	0	0	0	100
Total	192	96	95	0	1	0	99.5

The table reports: the number of samples tested, the number of individuals correctly sexed for males, the number of individuals correctly sexed for females, the number of false negatives (male sexed as female), the number of false positives (female sexed as male) shown in bold, the number of cases for which the PCR failed, and the true positive success rate.

The primers have been tested only with hemp-type cultivars. We explored whether the PCR multiplex mix could be used in drug-type cultivars of *C. sativa*. To do so, we ran an in silico amplification using the *Primer-BLAST tool* in the nr database (Sayers *et al*. 2021) (see https://www.ncbi.nlm.nih.gov/tools/primer-blast/index.cgi). We selected the genomes for which the sex was reported, and we used the default parameters for the amplification. A total of 8 female genomes and 3 male genomes were available for *C. sativa* (see Supplementary Table 1). The primers for autosomal locus #9, our internal control, successfully produced the expected size amplification for all genomes. None of the female genomes resulted in the amplification of any Y-specific primers. The sex of the 3 male genomes (including 2 drug type and 1 hemp type) was correctly identified by primer #23. However, the primer #36 was successfully amplified only in the hemp-type genome. This suggests that at least one of the 2 Y-specific primers could be effective in identifying the sex in drug-type cultivars of *C. sativa*.

## Discussion

In the present study, we developed a PCR multiplex mix, which includes 2 Y-specific markers and 1 autosomal marker to identify the sex of *C. sativa* samples. The multiplex mix correctly identifies the sex in 99.5% of the cases, which makes it among the most accurate methods of the Cannabaceae family (Toth *et al*. 2020; Clare *et al*. 2024). Only 1 female sample was identified as a male. Given that the sex was correctly assigned for 15 males and 14 females of the same cultivar, we cannot rule out the hypothesis of an error in the phenotypic identification of the sex or maybe a sex reversion of the individual (genotypic male with a female phenotype). Furthermore, our method offers a 100% specificity (i.e. absence of males assigned as females), which is crucial to avoid males in crops.

Although we only tested the mix in hemp-type cultivars, the in silico analysis showed that one of the 2 Y-specific markers amplified in drug-type genomes was obtained from male individuals. The in silico analysis relies on the quality of the Y assemblies, and it is possible that the other Y-specific markers did not amplify because of assembly errors. This suggests that the mix could be efficient to identify the sex of most, if not all, *C. sativa* cultivars. Future work should aim at testing our markers in drug-type cultivars.

## Supplementary Material

jkaf077_Supplementary_Data

## Data Availability

All the data are presented in the main text and Supplementary materials. Supplemental material available at G3 online.
