# Supplementary material for: Promising high fidelity genetic markers for sexing Cannabis sativa seedlings
Source: G3 (Bethesda). 2025 Apr 9;15(6):jkaf077. doi: 10.1093/g3journal/jkaf077 (PMC12135001; doi:10.1093/g3journal/jkaf077)
Supplement: jkaf077_Supplementary_Data [file jkaf077_supplementary_data.zip › Supplementary_Material_Legend_G3-2024-405539.docx]

**Supplementary information**

Supplementary Table 1: *In silico* PCR validation on hemp-type and drug-type genomes.
